# Molecular Analysis of Goodpasture’s Disease Following Hematopoietic Stem Cell Transplant in a Pediatric Patient, Recalls the Conformeropathy of Wild-Type Anti-GBM Disease

**DOI:** 10.3389/fimmu.2019.02659

**Published:** 2019-11-14

**Authors:** Paul E. Gray, Hugh McCarthy, Owen M. Siggs, Moin A. Saleem, Tracy O' Brien, Katie Frith, John B. Ziegler, A. Richard Kitching, Agnes B. Fogo, Billy G. Hudson, Vadim Pedchenko

**Affiliations:** ^1^Department of Immunology and Infectious Diseases, Sydney Children's Hospital, Sydney, NSW, Australia; ^2^Faculty of Medicine, School of Women's and Children's Health, University of New South Wales, Sydney, NSW, Australia; ^3^Department of Nephrology, Sydney Children's Hospital, Sydney, NSW, Australia; ^4^Immunology Division, Garvan Institute of Medical Research, Darlinghurst, NSW, Australia; ^5^Faculty of Health Sciences, Bristol Medical School, University of Bristol, Bristol, United Kingdom; ^6^Kid's Cancer Centre, Sydney Children's Hospital, Sydney, NSW, Australia; ^7^Faculty of Medicine, Nursing & Health Sciences, Centre for Inflammatory Diseases, Monash University, Clayton, VIC, Australia; ^8^Department of Pediatrics, Vanderbilt University School of Medicine, Nashville, TN, United States; ^9^Department of Pathology, Microbiology and Immunology, Vanderbilt University School of Medicine, Nashville, TN, United States; ^10^Division of Nephrology, Department of Medicine, Vanderbilt University Medical Center, Nashville, TN, United States; ^11^Department of Biochemistry, Vanderbilt University School of Medicine, Nashville, TN, United States

**Keywords:** Goodpastures, glomerular basement membrane, conformeropathy, GvHD, anti-GBM, alloimmunity, autoimmunity

## Abstract

**Background:** Goodpasture's disease (GP) is mediated by autoantibodies that bind the glomerular and alveolar basement membrane, causing rapidly progressive glomerulonephritis with or without pulmonary hemorrhage. The autoantibodies bind neoepitopes formed upon disruption of the quaternary structure of α345NC1 hexamer, a critical structural domain of α345 collagen IV scaffolds. Hexamer disruption leads to a conformational changes that transitions α3 and α5NC1 subunits into immunogens, however, the trigger remains unknown. This contrasts with another anti-GBM disease, Alports' post-transplant nephritis (APTN), where the pathogenic alloantibody binds directly to native NC1 hexamer. The current report includes the first study of antigenic specificity and allo-incompatability in anti-GBM disease occurring after allogeneic haematopoietic stem cell transplant (HSCT).

**Results:** The anti-GBM antibodies were found to be directed predominantly against the E_A_ epitope of the α3 NC1 monomer of collagen IV and developed rapidly in patient serum reaching peak level within 5 weeks. Autoantibody binding to native α345NC1 hexamer was minimal; however, binding was greatly increased upon dissociation of the native hexamer. There were no polymorphic genetic differences between donor and recipient collagen IV genes which would be predicted to cause a significant NC1 conformational change or to provide a target for antibody binding. Both patient and donor possessed the Goodpasture's susceptibility HLA-allele DRB1^*^1501.

**Conclusions:** The current report includes the first in-depth study of allo-incompatability and antigenic specificity in anti-GBM disease occurring after allogeneic haematopoietic stem cell transplant (HSCT). No polymorphic genetic differences were identified between donor and recipient collagen IV genes which would be predicted to provide a target for antibody binding. Furthermore, autoantibody binding to native α345NC1 hexamer was minimal, increasing greatly upon dissociation of the native hexamer, resembling wild-type GP diseases and marking this as the first example of a post-HSCT conformeropathy.

## Introduction

Goodpasture's (GP) disease is mediated by autoantibodies, commonly known as anti-GBM antibodies, that target α345 scaffold of collagen IV of the kidney glomerular (GBM) and lung alveolar basement membrane causing rapidly progressive glomerulonephritis and pulmonary hemorrhage. In GP disease, the autoantibodies bind neoepitopes formed on α3 and α5 NC1 subunits upon disruption of the quaternary structure of native α345NC1 hexamer, a critical structural domain of α345 collagen IV scaffolds (1, 2). Hexamer disruption is concomitant with conformational changes that transitions subunits into immunogens. The pathogenesis of the ensuing anti-GBM nephritis contrasts with that of Alport's post-transplant nephritis (APTN). In APTN, α345 collagen IV scaffold is seen as foreign within the transplanted kidney, owing to its absence in the GBM of the recipient Alport patient. Of particular importance, while both auto- and alloantibodies target the same α345NC1 hexamer, they bind different epitopes (1). In GP disease, epitopes are formed after dissociation of hexamer subunits, whereas in APTN alloantibody epitopes are located on the surface of the α345NC1 hexamer (3, 4). The trigger for conformational changes that transition subunits into immunogens in GP disease remains unknown. We describe the first case of anti-GBM disease occurring post haematopoietic stem cell transplant where the pathogenesis including antibody specificity and the local determinants of antibody binding have been extensively studied.

We previously reported the first patient with X-linked lymphoproliferative (XLP) disease-associated cerebral vasculitis to successfully undergo a hematopoietic stem cell transplant (HSCT) (5). However, at day +169 post-transplant the patient developed anti-GBM disease, a complication rarely reported post-HSCT. This case presented a unique opportunity to investigate whether the anti-GBM nephritis phenocopied alloimmune response, as in APTN, or autoimmune response, as in GP disease. In addition, serum from this patient was available prior to and during the onset of anti-GBM disease, providing a window into both the emergence, evolution, antibody specificity, and the temporal relationship between antibody production and the development of renal failure.

## Materials and Methods

Patient serum was collected prior to the onset of anti-GBM disease and through the time when plasma exchange and immunosuppressive drug treatment were initiated. Serum collected at the disease onset at dilutions of 1:50 to 1:250 was applied to frozen sections of normal human kidney, and binding of circulating anti-GBM antibody to the normal GBM was detected using fluorescein labeled anti-IgG secondary antibody. For immunoadsorbtion, patient serum was preincubated with NHS-activated magnetic beads coated with 100 μg/mg of recombinant α3NC1 domain of collagen IV. In a separate experiment, immunofluorescent staining was also performed after treatment of normal kidney sections with 6 M urea in 0.1 M glycine-HCl, pH 2.2 for 10 min prior to addition of GP serum.

### Antigens and Anti-GBM ELISA

Recombinant human α1NC1 through α6NC1 domains of collagen IV in monomer form and α3/α1 chimeras were purified from culture medium of stably transfected human embryonic kidney (HEK) 293 cells using anti-FLAG agarose as previously described (6). α3/α1NC1 chimeras corresponding to the E_A_ and E_B_ epitopes of the α3NC1 domain were constructed by PCR mutagenesis as previously described (7). The native NC1 hexamers of collagen IV were isolated from bovine or human GBM after collagenase digestion and size-exclusion FPLC chromatography. Binding of antibody to recombinant NC1 domains or chimeras was performed using indirect enzyme-linked immunosorbent assay, with alkaline phosphatase-conjugated goat anti-human IgG or IgM (Sigma, 1:2,000) secondary antibody as described (1).

### Sequencing and Analysis

Next generation gene sequencing was performed by the Bristol genetics laboratory using the *HaloPlex* Target Enrichment System kit including all coding regions for a range of basement membrane associated genes. Analysis was focused specifically on the COL4A3, COL4A4, and COL4A5 genes to identify non-reference sequence variations (hg19) between donor and recipient, which were assessed using the Grantham score of physicochemical change.

### Statistical Analysis

The results for all quantitative experiments are reported as mean ± SD of three independent experiments. To determine differences between groups, we used analysis of variance with multiple groups comparison by Holm-Sidak method (SigmaStat) with *P* < 0.05 considered to indicate statistical significance.

## Results

A 12-year-old boy underwent unrelated cord blood transplant (UCBT) for X-linked lymphoproliferative (XLP) disease caused by a mutation c.96G>C in the *SH2D1A* gene. The patient's primary disease has been reported elsewhere regarding novel features of XLP, with presentation including cerebral vasculitis, aplastic anemia, acute respiratory distress syndrome, and arthropathy (5). Features of the transplant potentially pertinent to the current investigations include that an initial 6/6 HLA matched UCBT failed to engraft and he underwent a second transplant with a 5/6 matched UCBT, which engrafted with 100% donor chimerism. His main side effects during the acute phase of the transplant were BK virus-associated hemorrhagic cystitis with bladder perforation and a possible NK cell immune reconstitution syndrome, including bilateral pulmonary infiltrates. At 169 days post-transplant when he had been engrafted and well for some time, he presented with fever, hematuria and acute renal failure, and was identified as having anti-GBM antibodies on indirect immunofluorescence of serum and characteristic crescentic glomerulonephritis injury with direct linear GBM immunofluorescence staining for IgG on renal biopsy. He was treated with plasmapheresis for 1 month with initial 2nd daily exchanges, high dose corticosteroids and cyclophosphamide before having B-cell depletion with rituximab. He went into remission, becoming anti-GBM antibody negative, with residual moderate chronic kidney disease. He is currently well with a glomerular filtration rate of 43 ml/min/1.73 m^2^, with no proteinuria or hematuria.

**Figure 1:**
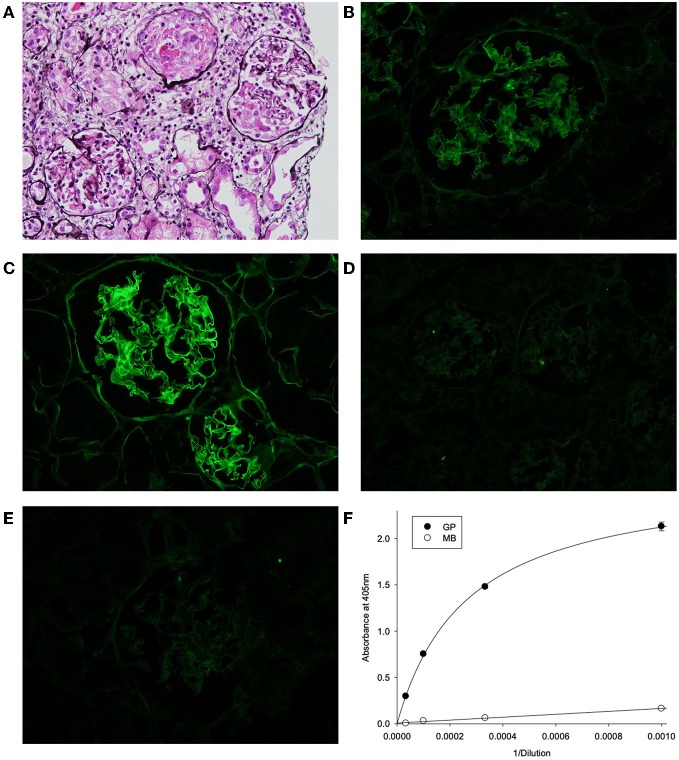
**(A)** Kidney lesions in post-HSCT patient showing characteristic features of crescentic glomerulonephritis, with >90% of the 32 glomeruli sampled displaying cellular or fibrocellular crescents, with segmental fibrinoid necrosis and with extensive acute tubular injury and focal, 10–20% interstitial fibrosis and tubular atrophy (Jones' silver stain). **(B–E)** Binding of patient serum antibodies to frozen sections from normal human kidney (immunofluorescent staining). **(B)** Distinct linear staining of GBM observed on intact kidney section, which is strongly increased after pre-treatment with acidic urea **(C)**. **(D)** There is no staining with normal human serum (1:50). **(E)** GBM staining was abolished by adsorption of patient serum on α3NC1-coated magnetic beads **(E)**, which removed 95% of α3-antibody as demonstrated by testing of original (GP) and absorbed (MB) serum using indirect ELISA of on α3NC1-coated plate **(F)**.

The biopsy showed characteristic features of crescentic glomerulonephritis, with >90% of the 32 glomeruli sampled (8 globally sclerosed) displaying cellular or fibrocellular crescents, with segmental fibrinoid necrosis and with extensive acute tubular injury and focal, 10–20% interstitial fibrosis and tubular atrophy (Figure 1A). When applied to frozen sections of normal human kidney, the patient's serum at 1:50 dilution demonstrated strong linear anti-GBM staining, which was greatly enhanced by acidic urea treatment (Figures 1B,C). The specificity of the staining and the nature of deposited antibody were established by immunoadsorbtion of serum on α3NC1-coated magnetic beads, which nearly abolished staining in parallel with removal of α3NC1 antibody (Figures 1E,F). The findings are diagnostic of severe anti-GBM antibody-mediated glomerulonephritis.

**Figure 2:**
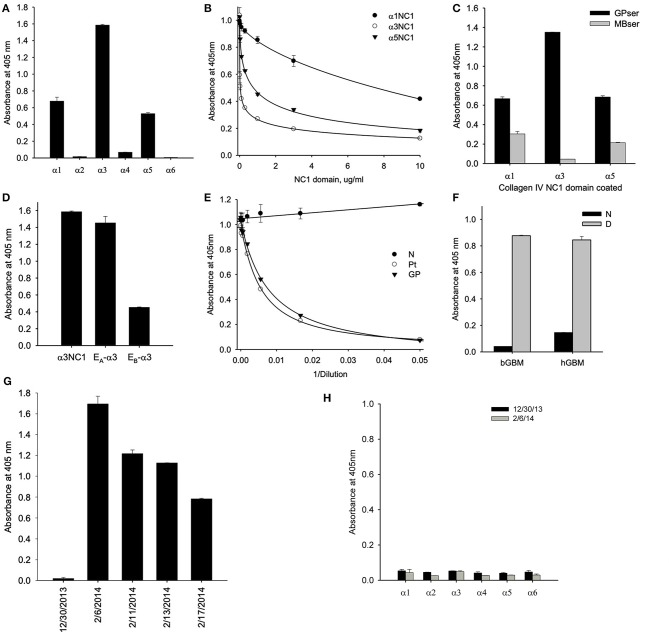
**(A)** Testing of NC1 domain specificity at the onset of the disease by ELISA showed that antibodies targeted predominantly α3NC1 domain with a lesser reactivity to α1 and α5NC1 domains. **(B)** Patient serum was pre-incubated with increasing concentrations of the α1, α3, and α5NC1 monomers and binding to corresponding immobilized monomers was measured by ELISA. Differential sensitivity to inhibition by the three NC1 monomers indicates that α3 antibodies has the highest affinity. **(C)** Pre-adsorption of patient serum with α3NC1-coated magnetic beads (MBser) significantly reduced immunoreactivity to α1 and α5NC1 monomers compared to original serum (GPser). **(D)** α3NC1-specific antibodies predominantly target the E_A_ epitope with lower reactivity to E_B_ epitope. **(E)** Binding of purified GP antibody labeled with biotin to α3NC1 monomer was competed by patient serum (Pt), as well as control serum mix from 8 GP patients (GP), but not by the normal human serum (N). **(F)** Patient's antibodies strongly react with the denatured NC1 hexamers from bovine (bGBM) or human (hGBM) glomerular basement membrane, while minimally react with native NC1 hexamers. **(G)** ELISA analysis of serial serum samples demonstrated the absence of anti-GBM antibodies 5 weeks prior to presentation (12/30/2013) by comaprison with normal human serum (NHS), with a rapid and strong development of immunoreactivity in 5 weeks. **(H)** There was no IgM binding to any of the six human recombinant NC1 domains of collagen IV in serum samples collected before or at the onset of the disease. The dashed line indicates threshold determined as mean ± 3x SEM for normal human serum.

Serum collected at initial presentation showed that a majority of antibody targeting the α3NC1 monomer of collagen IV with weaker reactivity against α1 and α5NC1 monomers, indicating that α3NC1 is the primary autoantigen (Figure 2A). This was further supported by measuring the affinity of circulating antibodies toward human α1, α3, and α5NC1 domains (Figure 2B). Patient serum was pre-incubated with increasing concentrations of the NC1 monomers and binding to immobilized α1, α3, and α5NC1, respectively was measured by inhibition ELISA. The strongest inhibition by the α3NC1 monomers indicates that the anti-α3 antibodies have highest affinity, followed by the anti-α5, and low affinity anti-α1 antibody. Furthermore, adsorption of patient serum on α3NC1-coated magnetic beads not only removes α3 specific antibodies, but significantly reduces immunoreactivity to α1 and α5NC1 (Figure 2C), indicating at least partial cross-reactivity of the major α3-specific antibody with the α1 and α5NC1 domains.

Circulating antibodies from the HSCT patient targeted predominantly the E_A_ epitope of α3NC1 monomer with lower reactivity toward E_B_ epitope (Figure 2D) suggesting a key role of the E_A_ region in the initiation of anti-GBM disease in this case. This is consistent with previously reported E_A_-specific antibody titers as a primary predictor of renal outcome in Goodpasture's disease (8) and proven pathogenicity of E_A_-α3 chimeric protein in rat model of glomerulonephritis (9). Since targeting of the additional non-GP epitopes could not be excluded in this type of assay, we performed the competition ELISA for antibody binding to the α3NC1 domain. For this experiment, GP antibodies were purified by affinity chromatography on α3NC1 domain coupled to agarose beads and labeled with biotin. Patient serum antibodies efficiently competed with binding of biotinylated GP antibody to coated α3NC1 in a dose-dependent manner and was undistinguishable from the control mix of GP sera (Figure 2E), showing that current patient's circulating antibody targets the same epitopes as in patients with classical GP disease.

Finally, although there was minimal antibody reactivity with native α345NC1 hexamers from bovine or human GBM, binding of antibody was significantly increased upon hexamer dissociation by a protein denaturant (Figure 2F). This closely resembled the enhanced immunofluorescent staining of human kidney sections after acidic urea (Figure 1C). Therefore, in terms of epitope specificity and binding to dissociated NC1 hexamers, the anti-GBM antibodies in the current post-HSCT case clearly phenocopy classical Goodpasture autoantibodies, but not APTN alloantibodies.

ELISA analysis revealed the rapid development of antibodies to the α3NC1 monomer of collagen IV in patient serum over a period of 5 weeks (Figure 2G). Surprisingly, we did not detect IgM reactivity to any of collagen IV NC1 domains either prior or or at the onset of the anti-GBM disease (Figure 2H). By quantitative analysis using purified GP antibodies as standard, we found that concentration of α3-specific IgG reached peak concentration about 400 μg/ml or 4% of total IgG, which is considerably higher than ~1% median value for GP patients.

Given that there is a strong association between susceptibility to Goodpasture's disease and HLA DRB1^*^15:01 (10–12), we also assessed the HLA alleles and found that both the recipient and the donor of the cord blood, which engrafted 100%, carried a single Goodpasture's associated HLA DRB1^*^15:01 allele.

We then questioned whether the pathogenesis of anti-GBM disease in this HSCT patient involved alloreactivity between the incoming donor immune system and genetic variation in the recipient's α345 collagen IV scaffold. An absence of genetic alterations within the part of the *COL4A3* gene encoding the NC1 domain in Goodpasture's patients has been reported previously, suggesting that NC1 mutations are not a major factor in the etiology of classical Goodpasture's disease (13). However, we hypothesized that genetic variations may occur in HSCT patient that alters the fine specificity of T and B cell tolerance to α3NC1 or the structure of the of the recipient's α345 NC1 hexamer. Such an alteration could promote appearance of the abnormally folded NC1 hexamer or an unassembled full length α3 chain recognized as foreign to the incoming donor immune system and thereby inducing antibody production.

**Figure 3:**
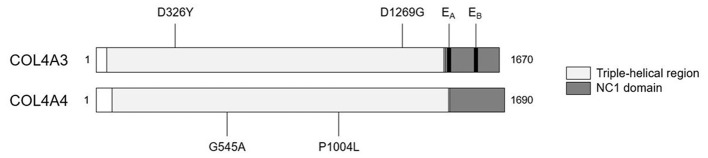
Patient-restricted coding variants within type IV collagen proteins. Domain architecture and relative locations of missense variants within COL4A3 (Uniprot: Q01955) and COL4A4 (Uniprot: P53420) proteins that differed between the patient and the cord-blood donor. No patient-restricted variants were observed in COL4A5. The black bars represent anti-GBM disease-associated E_A_-α3 and E_B_-α3 epitopes.

We detected two missense variants in each of *COL4A3* and *COL4A4*, and none in *COL4A5* genes in the recipient, but not in the donor, which are listed in Table 1. Mutation mapping shows that all four variants fall within the triple-helical region of *COL4A4*/*COL4A3* chains distant to the target E_A_-α3 and E_B_-α3 epitopes of the NC1 domain (Figure 3) and would not be predicted to directly influence the quaternary structure of the native α345NC1 hexamer. No variants were detected which differed between donor and recipient, in the α3, α4 or α5NC1 domains.

**Table 1:** Missense variants in genes encoding collagen IV chains.

**Variant**	**Gene**	**gnomAD MAF**	**SNP ref**.	**Amino acid change**	**Grantham score**
g.2,228163453,C,A	COL4A3	4.7%	rs57611801	Asp1269Glu	45
g.2,228121101,G,T	COL4A3	16.7%	rs55703767	Asp326Tyr	160
g.2,227946893,C,G	COL4A4	2.8%	rs1800516	Gly545Ala	60
g.2,227915832,G,A	COL4A4	52%	rs1800517	Pro1004Leu	98

*The gnomAD (r2.0.2) allele frequency (AF) reflects that these are common variants, however the Grantham scores (range 5–215) reveal that in particular rs55703767 (radical change), might be expected to impose a significant effect on the physicochemical properties of the α3 chain of collagen IV. MAF, minor allele frequency*.

## Discussion

Autoimmune disease occurring post-HSCT, i.e., antibodies from the graft against “self,” is not uncommon, and is more common with transplants are performed for certain monogenic indications (14). Wiskott Aldrich syndrome, a disease which predisposes to autoimmunity, is associated with a high rate of post-transplant autoimmunity, a predilection which is associated with reduced donor cell chimerism (15). HSCT for another entity, chronic granulomatous disease, may also predispose to a high risk of autoimmune cytopenias, neuropathy and thyroid disease (16), and in this case the tendency appears to occur despite excellent donor cell engraftment. There is no particular predilection reported of post-transplant autoimmunity in patients transplanted for XLP1.

The post-HSCT antibody-mediated disease where there is the greatest knowledge of antigenic target is autoimmune haemolytic anemia. Preformed recipient allo-antibodies against donor derived red cells resemble red cell alloantibodies important for transfusion reactions, and are therefore well-understood (17). However, there is little information of antigenic targets and in particular epitope specificities involved in less common post-HSCT allo- or autoimmune entities, with the exception of data on the cell surface targets of antibodies relevant to chronic graft vs. host disease (GVHD) (18).

The current patient is the first described case of anti-GBM disease post-cord blood transplant. Two cases of anti-GBM nephritis were reported originating as a graft-vs.-host (GVH) phenomenon post hematopoietic stem cell transplant (HSCT). A 42-year-old female patient received a bone marrow transplant (BMT) and developed systemic vasculitis with a combination of anti-neutrophil cytoplasmic (ANCA) and anti-GBM antibodies (19). In another study, a series of biopsies in patients suffering renal disease post-HSCT identified a single case of an elderly BMT patient with anti-GBM disease (20). Neither of these cases was analyzed with regard to antigen specificity of anti-GBM antibody.

Given the rarity of anti-GBM nephritis post-HSCT, we determined whether the specificity and binding properties of the anti-GBM antibodies of the HSCT patient resembled those of APTN or GP disease. This disease involved a donor immune system and genetically mismatched kidney, similar to the case with APTN, and therefore we had anticipated that the antibodies would behave analogous to that of APTN alloantibodies by binding to the native α345NC1 hexamer, and specifically to the α3 or α5NC1 subunits. Surprisingly, the HSCT antibodies did not bind the native α345NC1 hexamer but bound to the dissociated α3 and α5NC1 subunits via the E_A_ and E_B_ neoepitopes. Thus, the anti-GBM antibodies of the HSCT patient phenocopied GP autoantibodies. Furthermore, no genetic variants were found in the recipient collagen IV genes which might have led to a structural perturbation in the immunogen, eliciting antibody production.

We conclude that this is a case of classical Goodpasture's disease, as shown by the specificity of the antibodies phenocopying that of antibodies bound to native kidney and elicited by the native immune system of GP patients. These antibodies emerged post transplantation from a donor immune system. But what caused this patient to suffer this novel complication? The properties of his circulating antibodies, which were non-reactive to the native α345NC1 hexamer, but fully reactive to dissociated α3NC1 subunit, strongly suggest that the inciting immunogen was the dissociated subunit harboring the E_A_ and E_B_ neoepitopes. This supposition is supported by the finding that native α345NC1 hexamer is not pathogenic in an animal model of GP disease, whereas immunization with hexamer dissociated by acid treatment induced glomerulonephritis (21, 22). Yet, in the unique case of APTN, the native hexamer elicited alloantibody production, revealing it to be the immunogen in the kidney allograft (3, 4). While it is not possible to pinpoint the inciting factor that triggered hexamer dissociation into immunogenic subunits in the HSCT case, several possibilities exist. For example, after transplant he suffered an episode of bilateral pulmonary infiltrates, which could cause structural damage to the α345NC1 hexamer in the alveolar basement membrane (23), transitioning subunits into immunogens. Such a structural damage in lungs could also occur due to a bacterial or one of his viral infections (BK, HHV-7) or by the immunosuppression therapy. Indeed, lung injury has been associated with GP disease etiology, wherein cigarette smoking and inhalation of hydrocarbons are risk factors in numerous patients (24, 25).

The process of immune reconstitution may be relevant for GP disease etiology in HCT patients. The current paradigm suggests that the loss of tolerance in autoimmune disease is often a failure of multiple checkpoints that ultimately unmask autoreactive T and B cells (26). There have been reports of anti-GBM disease occurring with recovery following alemtuzemab treatment (27). Further, patients with HIV/AIDS, who have severe immune dysregulation, develop frequent autoantibodies, including anti-GBM, although anti-GBM disease is very rare (28). Both recipient and donor were homozygous for the HLA allele associated with Goodpasture's disease, HLA DRB1^*^15:01 (DR15) (11, 12). Homozygosity at the DR locus meant that there was no expression of dominantly protective DR allomorphs such as DR1 or DR7 (10), setting the scene for the loss of tolerance to α3NC1 under unfavorable conditions. In the context of DR15, central tolerance to α3NC1 is poor, as CD4^+^ Foxp3^−^ conventional T cells even from healthy people bearing DR15 react with the immunodominant T cell epitope α3_135−145_, and can be differentiated into effector Th1 cells, Th17 cells and T follicular helper (Tfh) cells (12). Antigen-specific Tfh cells are critical in generating antibody responses to most antigens, likely including the Goodpasture antigen. As the Tfh subset is deficient in *SH2D1A* deficiency (29), in our patient aberrant or exuberant reconstitution resulting in the development of autoreactive Tfh cells may have played a role in loss of tolerance to α3NC1 and the generation of anti-GBM antibodies.

The availability of serum prior to and during early stages of anti-GBM disease in this HSCT patient provided a unique opportunity to assess the time frame for development of GP disease. The case demonstrated the rapidity, in a matter of weeks, with which a patient can go from being antibody negative to accumulating high titers of pathogenic antibody with development of rapidly progressive glomerulonephritis. Importantly, our findings reiterate the ultimate importance for early diagnosis and timely treatment (30) with potential recovery of kidney function despite very high level of circulating α3 and α5NC1 antibodies. Furthermore, our patient shows that the antibody phenocopies the autoimmune GP disease, rather than the antibody specificity seen in Alport patients with anti-GBM developing after kidney transplant.

## Data Availability Statement

All datasets generated for this study are included in the article/supplementary material.

## Ethics Statement

This study has been approved by the Human Research Ethics Committee of the South Eastern Sydney Local Health Network—Northern Sector HREC Ref 11/107, and written informed consent obtained from the parents of the participant for the publication of this case.

## Author Contributions

PG, KF, and TO'B managed the patient. TO'B contributed regarding post-transplant autoimmunity. KF regarding immune reconstitution. PG and JZ envisaged the project. PG liaised with VP who performed the serological and staining work with BH and AF. PG and HM worked with MS who performed genetics in Bristol and OS helped with data interpretation, while AK advised regarding HLA testing and interpretation. PG and VP worked together to bring the manuscript together with the help of other investigators.

### Conflict of Interest

The authors declare that the research was conducted in the absence of any commercial or financial relationships that could be construed as a potential conflict of interest.
